# Application of PPAR Ligands and Nanoparticle Technology in Metabolic Steatohepatitis Treatment

**DOI:** 10.3390/biomedicines12081876

**Published:** 2024-08-16

**Authors:** Hung Thai Vu, Vien Duc Nguyen, Hiroko Ikenaga, Tsutomu Matsubara

**Affiliations:** 1 Department of Anatomy and Regenerative Biology, Graduate School of Medicine, Osaka Metropolitan University, Osaka 545-8585, Osaka, Japan; si22502u@st.omu.ac.jp (H.T.V.); sv22916q@st.omu.ac.jp (V.D.N.); 2 Department of Hepatology, Graduate School of Medicine, Osaka Metropolitan University, Osaka 545-8585, Osaka, Japan; 3 Research Institute for Light-induced Acceleration System (RILACS), Osaka Metropolitan University, Sakai 599-8570, Osaka, Japan

**Keywords:** PPAR, MASLD, MASH, nanoparticles, drug delivery

## Abstract

Metabolic dysfunction-associated steatotic liver disease/steatohepatitis (MASLD/MASH) is a major disease worldwide whose effective treatment is challenging. Peroxisome proliferator-activated receptors (PPARs) belong to the nuclear receptor superfamily and function as ligand-activated transcription factors. To date, three distinct subtypes of PPARs have been characterized: PPARα, PPARβ/δ, and PPARγ. PPARα and PPARγ are crucial regulators of lipid metabolism that modulate the transcription of genes involved in fatty acid (FA), bile acid, and cholesterol metabolism. Many PPAR agonists, including natural (FAs, eicosanoids, and phospholipids) and synthetic (fibrate, thiazolidinedione, glitazar, and elafibranor) agonists, have been developed. Furthermore, recent advancements in nanoparticles (NPs) have led to the development of new strategies for MASLD/MASH therapy. This review discusses the applications of specific cell-targeted NPs and highlights the potential of PPARα- and PPARγ-targeted NP drug delivery systems for MASLD/MASH treatment.

## 1. Introduction

Fatty liver disease, also known as hepatic steatosis, is a major chronic liver disease affecting approximately 25% of the global population. It is projected to become the primary indication for liver transplantation by 2030, thus placing a significant burden on global health [1,2,3,4,5,6,7,8,9]. Fatty liver is commonly classified into two types based on alcohol intake history: metabolic dysfunction-associated steatotic liver disease/steatohepatitis (MASLD/MASH) and alcoholic liver disease [3,4,5,6,7,8,9,10,11]. Alcoholic liver disease is characterized by liver fat accumulation due to substantial alcohol intake, whereas MASLD is a broad term encompassing various liver conditions with varying degrees of damage and fibrosis [3,5,12,13]. Hepatic steatosis is a feature of MASLD that is characterized by the presence of fat in the liver, making up 5–10% of its weight. MASH involves inflammation and fibrosis, which can further progress to cirrhosis and hepatocellular carcinoma (HCC) [14,15,16]. Approximately 20% of patients with MASLD progress to MASH, and over 40% of those with MASH develop fibrosis [16,17]. Dysregulation in lipid and lipoprotein metabolism, chronic inflammation, and oxidative stress are the primary risk factors that play critical roles in the pathogenesis of MASLD/MASH [18]. Despite significant research efforts, the mechanisms underlying MASLD/MASH remain unclear. The traditional “two-hit” hypothesis is now considered insufficient to fully explain the complex molecular and metabolic changes associated with MASLD/MASH. In contrast, the “multiple hit” hypothesis offers a more comprehensive framework to describe the interplay among various factors, including insulin resistance (IR), adipose tissue hormone levels, nutrition, gut microbiota, and genetic and epigenetic factors. Pathogenesis of MASLD/MASH is a complex and multifaceted process involving several interconnected factors [19]. 

Peroxisomes, originally known as microbodies, were discovered as intracellular organelles by de Duve et al. in 1960 [20]. They are mainly involved in respiration via the H_2_O_2_ system and β-oxidation of FAs, along with other metabolic activities [21]. Peroxisomal proliferation is stimulated by various compounds, such as clofibrate and di[2′-ethylhexyl] phthalate. Peroxisome proliferator-activated receptors (PPARs) mediate peroxisomal proliferation. In the 1990s, PPARs became members of the nuclear receptor superfamily of ligand-activated transcription factors in rodent hepatocytes. PPARs play critical roles in the control of lipid metabolism via the regulation of transcription of genes involved in FA, bile acid, and cholesterol metabolism [22]. To date, three distinct subtypes of PPARs have been characterized: PPARα, PPARβ/δ, and PPARγ. PPARα and PPARγ have been extensively studied in the context of MASLD/MASH. PPARα is mainly expressed in the liver and brown adipose tissue. It plays a crucial role in regulating the expression of genes responsible for FA transport and oxidation, which are vital for maintaining lipid homeostasis [23]. In contrast, PPARγ is mainly expressed in the adipose tissue, where it regulates adipocyte differentiation and insulin sensitivity. It performs various biological functions, such as fat and glucose metabolism regulation, tumor cell differentiation, and apoptosis induction. PPARγ also promotes ovulation, exhibits anti-atherosclerotic activity, improves heart failure and ventricular remodeling, and inhibits inflammation [24]. 

The currently available treatments for MASLD/MASH exhibit suboptimal safety and efficacy. Despite the availability of various therapeutic approaches, including insulin sensitization, antioxidant pharmacotherapy, and lifestyle interventions, such as weight loss and bariatric surgery, no United States Food and Drug Administration-approved treatment is available for MASLD/MASH [25,26]. Efficient cell-specific drug delivery has immense potential to overcome the limitations of current therapeutic strategies by enhancing the efficacy of targeted agents while minimizing the off-target effects [27]. Nanomedicine is a promising solution that leverages the cell-specific targeting potential of nanoparticles (NPs). Nanomedicine emerged based on the seminal work of Robert A. Freitas in 1999. Its primary goal was to create nanoscale materials and devices capable of revolutionizing medical applications [28]. The global research and development landscape in nanomedicine is vibrant, with major contributions from regions such as the United States, the European Union, and Japan. Overall, nanomedicine shows significant potential for the development of highly specific and effective therapeutics for chronic metabolic liver diseases. By harnessing the precision and versatility of nanotechnology, effective liver disease treatment can be achieved with improved therapeutic outcomes.

MASLD/MASH progresses via lipid accumulation (hepatocytes) [22], inflammation (inflammatory cells, such as macrophages) [29], and fibrosis (hepatic stellate cells [HSCs]). Determination of the pathogenesis of MASLD/MASH is necessary to identify the molecular changes in liver cells. In this review, we summarize the roles of PPARα and PPARγ in MASLD/MASH and discuss the potential of NP technology for the development of specific cell-targeting drug delivery systems to enhance the therapeutic outcomes of liver diseases, including MASLD/MASH.

## 2. Lipid Homeostasis in the Liver

Hepatic lipid homeostasis is mainly regulated by the uptake, synthesis, degradation, and excretion of FAs in hepatocytes [30]. Glucose and insulin promote FA synthesis (lipogenesis) via synergistic effects of sterol regulatory element-binding protein 1C (SREBP-1C) and carbohydrate response element-binding protein (ChREBP) [31,32]. Insulin induces SREBP-1C expression via alpha serine/threonine-protein kinase signaling and enhances SREBP-1C activity. Activation of hepatic SREBP-1C results in the regulation of lipogenic genes, including fatty acid synthase (FAS) and acetyl-CoA carboxylase (ACC) [33,34]. Elevated glucose levels induce the phosphorylation-dependent nuclear relocalization of ChREBP, inducing *FAS* and *ACC* expression [35]. In addition to de novo synthesis, hepatic lipid stores consist of FAs derived from the breakdown of triglycerides (TGs; lipolysis) in adipose tissues and dietary intake [36]. FAs are transported to hepatocytes via fatty acid transport protein, fatty acid translocase, and cluster of differentiation (CD) 36, which facilitates their uptake. Upon the entry of FAs into hepatocytes, they bind to the fatty acid binding protein 1 (FABP1) and undergo peroxisomal β-oxidation by acyl-CoA oxidase 1 or are shuttled into mitochondria by the carnitine-palmitoyl transferase/carnitine-acyl carnitine carrier system before undergoing β-oxidation. In mitochondria, medium-chain acyl-CoA dehydrogenase and long-chain acyl-CoA dehydrogenase primarily mediate FA β-oxidation [37]. When β-oxidation can no longer process FAs, acyl CoA cholesterol acyltransferases (ACAT1 and 2) facilitate the esterification of FAs to cholesteryl esters (CEs) [38], whereas diacylglycerol-O-acyltransferases (DGAT 1 and 2) facilitate their esterification to TGs in the endoplasmic reticulum (ER) [39,40], which become the content of very-low-density lipoprotein (VLDL; secreted from hepatocytes via the Golgi apparatus) and lipid droplets (accumulated in hepatocytes). DGAT1 primarily localizes to the ER and catalyzes the formation of TGs using FAs derived from dietary sources [39,40]. In contrast, DGAT2 translocates from the ER to lipid droplets [39,40] and promotes TG formation using de novo-synthesized FAs [40]. Hepatocytes perform lipolysis and autophagy to recruit FAs for mitochondrial/peroxisomal β-oxidation or VLDL secretion. The assembly of FAs for VLDL formation is derived from the reesterification of TGs stored in lipid droplets [41]. In various cell types, adipose triglyceride lipase (ATGL) and its cofactor, comparative gene identification (CGI)-58, along with hormone-sensitive lipase and monoacylglycerol lipase, participate in the cleavage of FAs from TGs [42,43]. However, in hepatocytes, the contribution of these lipases to lipolysis remains unclear owing to the low expression of c in the human liver and lack of significant changes in hepatic VLDL secretion upon overexpression or knockdown of ATGL, hormone-sensitive lipase, and monoacylglycerol lipase [44]. Consequently, other lipases, such as TG hydrolase, in the ER contribute to hepatic VLDL formation. Liver-specific deletion of *Atgl* or *Cgi-58* in mice leads to steatosis and fibrosis due to enhanced hepatic β-oxidation, suggesting that these lipases mobilize lipids for β-oxidation [42,44]. 

## 3. Pathogenesis of MASLD/MASH

Fatty liver is associated not only with IR and disturbances in fat metabolism but also with various biological processes, including glucometabolic disorder, oxidative stress, and intracellular inflammatory response. Notably, these processes are interrelated and synergistically contribute to MASLD [45]. 

High FA levels trigger the increase in lipid peroxidation in the liver, and elevated lipid peroxidation leads to persistent production of reactive oxygen species (ROS) [46]. In addition to the known factors associated with increased ROS levels, other factors, such as inflammatory cytokines, adipokines, endotoxins, and mitochondrial inactivation, also boost lipid peroxidation in the liver [19]. Oxidative stress induced by the accumulation of FAs ultimately leads to the progression of MASLD, which promotes inflammation and cell death. Inflammation is positively correlated with liver injury and negatively correlated with hepatic lipolysis and stimulates lipid peroxidation, exacerbating the pathogenesis of MASLD/MASH [47]. ER stress is another crucial factor for the development of MASLD/MASH [48,49]. Metabolic dysfunctions, such as obesity and diabetes, induce ER stress, which results in the build-up of unfolded protein responses and impairs the proper physiological functioning of hepatocytes [50]. ER stress triggers the activation of SREBP-1C, thereby promoting the transcription of *FAS* and *ACC* and resulting in increased FA synthesis and lipid droplet formation in the liver [51]. Considering that lipid peroxidation can also induce ER stress and FA accumulation in hepatocytes, it is considered an important factor in aggravating MASLD/MASH [52]. Increased lipid accumulation during MASLD/MASH can lead to a microbial imbalance, resulting in the infiltration of bacteria and their products into the bloodstream and increasing the lipopolysaccharide (LPS) concentration in the liver [53,54,55]. Upon excess LPS condition, pro-inflammatory cytokines, including tumor necrosis factor-alpha (TNF-α), interleukin (IL)-1β, IL-6, IL-8, and C–C motif chemokine ligand 2, and ROS, are produced, and then activate Kupffer cells (KCs) via the toll-like receptors (TLRs) [56,57,58]. Activated KCs further trigger signals CD14, myeloid differentiation primary response 88, myeloid differentiation factor 2, mitogen-activated protein kinases (c-Jun N-terminal kinase), and nuclear factor kappa B (NF-κB), thus promoting inflammation. KC activation by IL-4 and IL-13 promotes tissue remodeling and the immune regulatory functions of macrophages. Therefore, the change between pro-inflammatory (M1) and anti-inflammatory (M2) phenotypes is an essential mechanism in the regulation of inflammatory responses [59,60].

**Figure 1:**
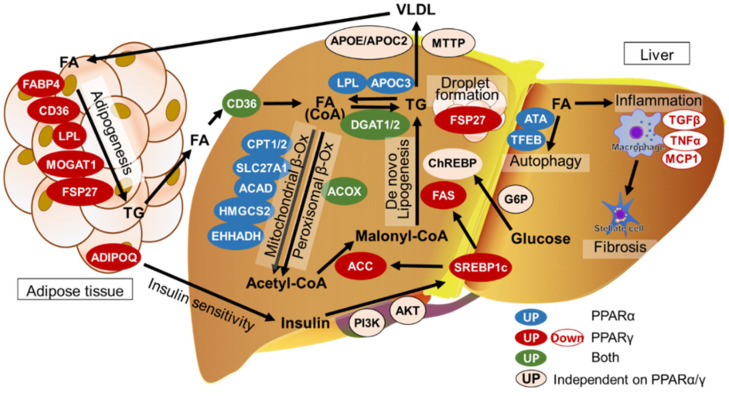
PPAR targets and contributing factors in MASLD/MASH. C1Q and collagen domain containing adiponectin (ADIPOQ) is a key insulin regulator whose secretion from the adipose tissue into the liver is regulated by PPARγ. Insulin regulates SREBP-1C via the PI3K/AKT signaling pathway in the liver. PPARα regulates the essential glycolytic enzymes ACC and FAS that play key roles in the synthesis of TGs. ChREBP is also involved in TG synthesis. LPL and APOC3 promote TG hydrolyzation into FAs. CPT1/2, SLC27A1, ACAD, HMGCS2, and EHHADH are involved in mitochondrial β-oxidation. In the presence of ACC, acetyl-CoA is converted to malonyl-CoA (de novo lipogenesis). Peroxisomal β-oxidation is enhanced via ACOX and DGAT1/2. Excess TG is transported to adipose tissue by VLDL facilitated by the expression of apolipoproteins, APOE and APOC2, and MTTP. In adipose tissues, FAs are converted into TGs via FABP4, CD36, LPL, MOGAT1, and FSP27. FA accumulation can cause autophagy controlled by ATA and TFEB. In MASH, downregulation of TGF-β, TNF-α, and MCP1 levels triggers hepatic inflammation, which subsequently activates the hepatic stellate cells and can lead to fibrosis.

Figure 1 shows the relationship between FA homeostasis in the liver and PPARα/γ levels. The functions of PPARα/γ and homeostasis of FAs in the liver are described below.

## 4. Single-Nuclei RNA Sequencing of PPARα and γ Expression Levels in Hepatic Cells

**Figure 2:**
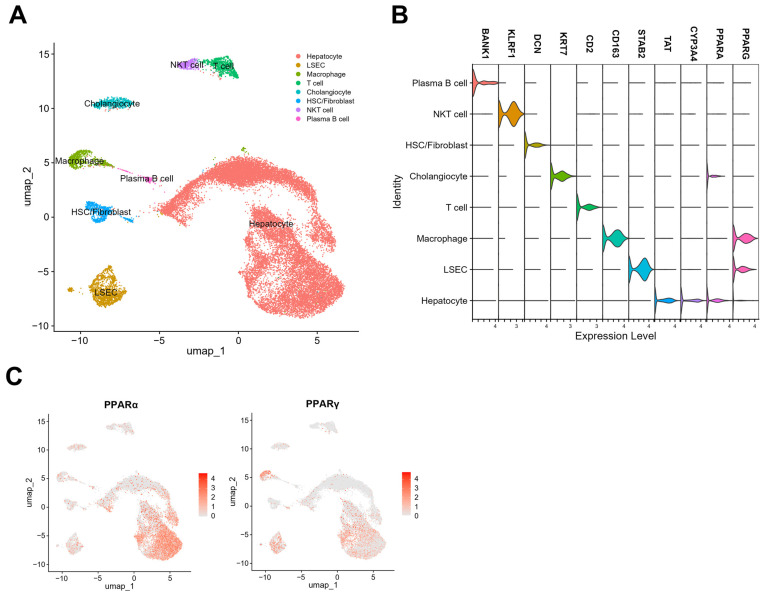
Single-nuclei RNA sequencing of PPARα and -γ expression levels in human hepatic cells. (**A**) Uniform manifold approximation and projection (UMAP) plots show the annotations for different cell types. (**B**) Violin plots of PPARα and PPARγ expression levels with marker genes for nine distinct cell types. (**C**) Feature plots. **Abbreviations:** BANK1, B cell scaffold protein with ankyrin repeats 1; KLRF1, killer cell lectin receptor F1; DCN, decorin; KRT7, keratin 7; STAB2, stabilin 2; TAT, tyrosine aminotransferase; CYP3A4, cytochrome P450 family 3 A member 4. **Methods:** Gene expression patterns of hepatic cells were investigated via single-nucleus RNA-sequencing in healthy individuals and patients with MASLD. Data were obtained from the Gene Expression Omnibus (GEO; accession number GSE174748). The Seurat package (version 5.0.1) was used to analyze the single-cell RNA sequencing data [63]. Filtering was used to remove the cells with ovecellsochondrial genes or fewer than 200 genes. Cells were normalized and clustered using Seurat. The samples were integrated using a harmony approach [64]. Cell types were annotated based on the expression levels of specific gene markers. UMAP, features, and violin plots were generated using the R statistical software (version 4.3.2).

Lipid homeostasis is an important process for liver function. PPARα and -γ regulate various processes, such as FA synthesis and oxidation, insulin sensitivity, and inflammatory factor levels in the liver [61]. A recent study determined the PPARα and PPARγ expression levels in the liver via single-nuclei RNA sequencing (Figure 2). PPARα and PPARγ expression levels were reanalyzed using a data set [62], revealing the highest PPARα levels in hepatocytes and cholangiocytes and PPARγ levels in macrophages, liver sinusoidal endothelial cells (LSECs), and hepatocytes in mice.

## 5. PPARα Functions in Hepatocytes, Cholangiocytes, and Macrophages

PPARα functions as an intracellular lipid sensor in hepatocytes that is activated by various endogenous or naturally occurring biological molecules, including various FAs and FA derivatives, such as acyl-CoAs, oxidized FAs, phytanic acids, endocannabinoids, and eicosanoids [65]. During fasting, PPARα functions as a key regulator of hepatic lipid metabolism by promoting the transportation and oxidation of FAs, as well as ketogenesis. This is attributed to the ability of PPARα to act as a master regulator. Specifically, PPARα was found to upregulate CD36, which facilitates the uptake of FAs into hepatocytes, and FABP1, which enables their transfer into the mitochondria for oxidation [37]. Furthermore, PPARα stimulates the expression of genes associated with mitochondrial FA β-oxidation, such as medium-chain acyl-CoA dehydrogenase and long-chain acyl-CoA dehydrogenase [65]. Additionally, PPARα upregulates the expression of genes involved in the biosynthesis of ketone bodies, including mitochondrial 3-hydroxy-3-methylglutaryl-CoA synthase. This critical ketogenic enzyme catalyzes the condensation of acetyl-CoA and acetoacetyl-CoA to produce 3-hydroxy-3-methylglutaryl-CoA, which is the initial ketone body [37]. Therefore, PPARα contributes to the decrease of FA levels in the liver and reduces lipid peroxidation and ROS production via enhancement of mitochondrial β-oxidation and ketone body generation, thus resulting in the protection of the liver from lipotoxicity. Notably, PPARα is activated by a carbohydrate-rich diet and stimulates lipogenesis by promoting SREBP-1C transcription [37]. SREBP-1C induces lipogenic genes, such as *FAS* and *ACC* [51]. In addition, PPARα indirectly controls SREBP-1C activity by inducing the expression of liver X receptor that regulates *SREBP-1C* gene transcription [66]. PPARα promotes the expression of lipoprotein lipase (LPL), an enzyme that catalyzes the breakdown of plasma TGs within lipoproteins. PPARα also induces the expression of genes involved in the production of apolipoprotein A1 (APOA1) and A2 (APOA2), which are the principal proteins in high-density lipoproteins [37,67]. PPARα also influences lipoprotein metabolism by reducing hepatic VLDL synthesis, leading to an increase in high-density lipoprotein levels. In hepatocytes, PPARα acts as a metabolic switch between fasting and postprandial states and is a part of the TG/FA cycle [37]. 

PPARα plays crucial roles in the reduction of oxidative stress, attenuation of cholestatic liver injury, and maintenance of bile acid homeostasis by regulating bile acid biosynthesis [68]. To date, only a few studies have investigated the alteration of PPARα expression in cholangiocytes. PPARα expression in cholangiocytes is decreased in cirrhotic primary biliary cholangitis livers, accompanied by increased microRNA (miR) 21 expression levels. As the overexpression of miR-21 favors the adhesion of inflammatory cells [69], the altered PPARα expression in cholangiocytes may lead to the progression of disease via changing cholangiocyte function [70].

Although the single-nuclei RNA sequencing showed low expression in macrophages, many previous studies have determined that PPARα plays a crucial role in the regulation of cholesterol and FA homeostasis in macrophages [71]. PPARα decreased the macrophage’s absorption of glycated LDL, a subtype of proatherogenic particles that carry glycated apolipoprotein (apo) B, in patients with diabetes. More interestingly, PPARα promotes LPL gene expression and suppresses its secretion and enzyme activity at the same time. In addition, recent studies demonstrated that PPARα can enhance intracellular lipid transport and metabolism in human macrophages [71,72]. As a result, PPARα activation led to the post-lysosomal mobilization of cholesterol via altering the expression of Niemann Pick type C 1 and 2, which contributes to the accumulation of cholesterol in the plasma membrane [73]. 

Moreover, PPARα activation decreases CE levels by increasing CPT1, which leads to a decrease of FAs as a substrate for ACAT1. Also, PPARα could affect CE hydrolysis via enhancement of neutral CE hydrolase gene expression [74,75,76]. PPARα is also a regulator of genes that are involved in the cholesterol efflux pathway. In human macrophages, PPARα contributes to the upregulation of ATP binding cassette subfamily A member 1 expression via indirect mechanisms that participate in the induction of liver X receptor alpha expression. This results in an increase in the APOA1-mediated cholesterol efflux back into the liver [77]. In summary, PPARα plays a key role in the regulation of cholesterol homeostasis in macrophages; however, its action mechanisms remain unclear, warranting further investigation. Moreover, owing to the significant differences between human and murine macrophages, cholesterol homeostasis regulation may occur in a species-specific manner [73].

## 6. PPARγ Functions in Macrophages, LSECs, and Hepatocytes

Expression of the PPARγ suppresses the immunoreactive state of macrophages by decreasing nitric oxide synthase 2, TNF-α, IL6, IL1β, and Monocyte chemoattractant protein 1, and promotes immunotolerant factors, such as CD36, IL-13, Arginase 1, Ym1, Inflammatory zone 1, CD206, IL-4, and IL-10, in macrophages [78,79]. 

Moreover, the evidence that PPARγ inhibits hypoxia-inducible factor 1-alpha, the immune reactive phenotype, and promotes Arginase 1, the marker of the immune tolerant macrophage, expressions showed a crucial role of PPARγ in precise macrophage functions [80,81]. Therefore, during MASH progression, the M1/M2 ratio was used to elucidate the pro-inflammatory conditions.

PPARγ mainly works as a suppressor of inflammation in the livers of MASH. Macrophage-specific *Pparγ* deficiency (*Pparγ*ΔLyz2) showed that certain upregulated genes were enhanced in pathways related to NOD-like receptor signaling, NF-κB signaling, chemokine-signaling, and cytokine–cytokine receptor interaction, suggesting an amplification of inflammatory cytokine and chemokine production driven by NLR family of pyrin domain containing 3 (NLRP3) and NF-κB signaling [82,83]. Downregulated differentially expressed genes were enriched in the PPAR signaling pathway and IR, suggesting severe metabolic disorders. In addition, macrophage-specific *Pparγ* deficiency showed significantly increased expression of liver fibrosis-related genes, such as transforming growth factor-beta 1 (TGF-β1), actin alpha 2, collagen type I alpha 1 (COL1A1), and tissue inhibitor of metalloproteinase (TIMP)-1, in MASH liver. Removal of PPARγ from macrophages can impact macrophage phenotypes, which influence the activation of HSCs and the development of MASH [24,84]. HSCs are activated by the excess of some proteins in the extracellular matrix, such as COL1A, which accumulates in liver fibrosis.

An imbalanced immune response is the major cause of liver injury in patients with MASH. LSECs are non-parenchymal cells in the liver that are located between the systemic arterial and portal venous blood within the sinusoids and liver parenchyma, forming an extraordinary barrier between the bloodstream and the underlying hepatocytes and HSCs within the space of Disse. LSECs act as blood pressure regulators and powerful scavengers, providing infiltration in and out of necessary substances, such as metabolites, solutes, and waste products, between the blood and hepatocytes through the space of Disse. Moreover, LSECs are involved in the immune response by producing cell signals, such as the pro-inflammatory cytokine IL-6. According to single-nuclei RNA sequencing, PPARγ was also found to be expressed in LSEC, and activation of *Pparγ* in LSECs promotes the LPS-stimulated IL-6 reduction, emerging the anti-inflammatory functions. Similar to macrophages, activated PPARγ inhibited the production of interferon-γ in splenic T cells [85,86]. However, at the present moment, exact PPARγ functions in LSECs have not been determined yet, and in subsequent studies, the answer to this question is expected to be discovered in the future.

Under normal physiological conditions, the expression of PPARγ in the hepatocytes is minimal. However, its expression was induced during the onset of steatosis. PPARγ stimulates the accumulation of lipid droplets in hepatocytes by upregulating the expression of hepatic fat-specific protein 27 (Fsp27), which is a key player in the process of lipid droplet formation [61]. In addition, hepatocyte-specific *Pparγ* deletion improves hepatic steatosis by attenuating the expression of the de novo lipogenesis genes (*FAS*, *ACC*, and *SCD1*). PPAR*α* activation contributes to decreased lipid accumulation in the liver of patients with MASLD/MASH, although PPARα has the ability to induce the de novo lipogenesis genes. Based on the current understanding, it is believed that hepatic steatosis is primarily driven by the activity of hepatocyte PPARγ, as opposed to PPARα [87]. PPARγ is also a nuclear receptor that is activated by ligands and exerts potent anti-inflammatory effects [88]. PPARγ agonists exert anti-inflammatory effects by inhibiting the activation of the NLRP3 inflammasome in hepatocytes that are laden with lipids [89]. It has also been reported that a deficiency in PPARγ can lead to increased ROS production, activation of the NLRP3 inflammasome, and secretion of IL-1β in hepatocytes following exposure to FAs. Conversely, the manipulation of PPARγ activity was able to alleviate MASLD induced by a high-fat diet by regulating both lipid metabolism and oxidative stress in hepatocytes through the activation of the nuclear factor erythroid 2-related factor 2 [90]. PPARγ caused liver cells to accumulate more lipids, which reversed the polarization of macrophages to M1. It also reduced the activation of a pathway called TLR4/NF-κB, which is involved in inflammation. When *Pparγ* was deleted specifically in liver cells, it sped up the process of macrophages becoming pro-inflammatory and activated the TLR4/NF-κB pathway. Hepatocyte-specific *Pparγ* deletion has been shown to augment TNF-α-induced C-X-C motif chemokine ligand 1 production [84]. Based on these findings, it appears that hepatic PPARγ can work as a potent regulator of hepatic inflammation.

## 7. PPARγ Functions in HSCs

HSC activation is key in liver fibrosis. TGF-β is an important factor in various essential processes, especially in HSC activation, by inducing α-SMA expression. A relationship between PPARγ and TGF-β has been elucidated by using PPARγ agonists and antagonists. A study showed that a PPARγ antagonist GW9662 dramatically increases TGF-β and α-SMA, while a PPARγ agonist GW1929 decreases TGF-β and α-SMA in primary HSCs, turning their shape into circular-like morphology [91]. As expected, two other studies also showed that GW9662 increases the fibrotic gene expressions in human HSCs [92,93]. In addition, overexpression of PPARγ showed a significant reduction of α-SMA, TIMP-1, and TIMP-2 in methionine and choline-deficient (MCD) diet-fed mice [94]. An experiment using *Pparγ*-knockout quiescent HSCs (Lrat-Cre-driven Pparγ deletion) revealed a decrease in E26 transcription-specific transcription factor 1 that is involved in maintaining HSC quiescence, highlighting the role of PPARγ in HSCs [95]. Recently, autophagy has been determined to be involved in liver fibrosis. In LX-2 cells (immortalized HSCs), ATG7 regulates autophagy via increasing microtubule-associated protein 1 light chain 3B and transcription factor EB (TFEB) expression levels [96]. Under rosiglitazone treatment, PPARγ activation suppresses autophagy in HSCs by reducing microtubule-associated protein 1 light chain 3B and TFEB expression, resulting in a decrease of autophagic vesicles and restoring lipid droplet levels [96]. 

Overall, PPARγ maintains HSCs via regulating lipid homeostasis, cell proliferation, and autophagy, which are associated with the activation of HSCs [96,97,98]. Because PPARγ is significantly decreased in HSC activation, upon HSC activation, PPARγ maintenance may be a strategy to treat liver fibrosis.

## 8. PPARα and PPARγ Functions in Adipose Tissues

Specific roles of PPARα in adipose tissues are not fully understood as its roles overlap with those of PPARγ. Some studies have indicated that PPARα affects thermogenesis in brown adipose tissue and browning in white adipose tissue [99]. Elevated free FA levels and increased TAG synthesis in the liver are due to the breakdown of white adipose tissue via lipolysis. Notably, PPARγ is the “master regulator” of adipogenesis [100,101]. PPARγ is a well-characterized regulator of energy metabolism that improves the uptake and expenditure of FAs in adipocytes and plays key roles in the pathophysiology of obesity and related complications. Adipocyte *Pparγ* deletion is associated with IR in mice [99]. Furthermore, PPARγ activation increases the expression of adiponectin in adipocytes. Specific *Pparγ* deletion in adipocytes leads to severe lipoatrophy in mice. Adipocyte-specific *Fsp27* deletion or *Pparγ* deletion also inhibits lipid accumulation in mouse adipose tissue. Adipocyte-specific *Fsp27* deletion exacerbates hepatic steatosis following a high-fat diet. Therefore, PPARγ/FSP27 in adipocytes may be a key regulator for hepatic fatty homeostasis, whose activation suppresses the development of MASLD/MASH [102,103,104].

## 9. Clinical Trials and Drugs Used for MASH: Potency of PPAR Ligands as Therapeutic Agents

PPAR activation is promoted by FAs, eicosanoids, and phospholipids produced during the metabolism of FAs in cells or from dietary sources of lipids. PPARs are also activated initiatively by synthetic ligands, such as fibrate (PPARα ligand), thiazolidinedione (TZD; PPARγ ligand), glitazars (PPARγ ligand), and elafibranor (dual PPARα and β/δ ligand; Table 1). PPAR agonists are widely used in basic research, with many undergoing clinical trials as potential therapeutic agents for MASLD/MASH [105].

### 9.1. PPARα Agonists 

Studies using mouse models have shown that PPARα agonists, such as Wy-14643, can prevent the induction of hepatic TG accumulation via an MCD diet in wild-type mice, but they have no effect on *Pparα*-deficient mice [106,107]. The potential use of PPARα agonists, particularly fibrates, in MASH treatment has been of interest for over two decades [108,109]. Gemfibrozil can reduce the levels of enzymes, including aspartate aminotransferase, alanine aminotransferase, and gamma-glutamyl transferase, in the liver and VLDL in patients with MASH. Studies on patients with type 2 diabetes (T2D) and hypertriglyceridemia indicated that fenofibrate reduces the serum levels of the inflammatory marker RANTES, thus indicating its potential anti-inflammatory effects related to MASLD. However, a clinical trial found no histological improvement in liver enzyme levels after a 48-week fenofibrate treatment of MASH patients with confirmed MASLD via liver biopsy. Interestingly, pemafibrate displays the potential to ameliorate liver dysfunction in patients with T2D and improve MASH pathology in mouse and rat models by enhancing lipid metabolism and reducing inflammation [67,109,110,111].

### 9.2. PPARγ Agonists

The functions of PPARγ agonists have been widely investigated in studies usually employing TZDs (pioglitazone and rosiglitazone), which are a class of insulin-sensitizing drugs [112]. These drugs enhance the uptake and storage of FAs in adipose tissue, thereby protecting the skeletal muscles and liver. Rosiglitazone prevents MASH development in patients on an MCD diet [109]. However, the drug was withdrawn from the European market because of its association with a high risk of myocardial infarction and heart failure. A preclinical study employing mouse models indicated that pioglitazone displays anti-inflammatory and antifibrotic properties by inhibiting platelet-derived growth factor (PDGF) and tissue inhibitor of metalloproteinase-2 [107]. The PIVENS (Pioglitazone, Vitamin E, or Placebo for Nonalcoholic Steatohepatitis) trial evaluated the potential efficacy of pioglitazone/vitamin E for MASH therapy. In the PIVENS trial, long-term pioglitazone treatment resulted in the resolution of MASH in more than 50% of the patients with prediabetes or T2D [109]. However, pioglitazone is associated with an increased risk of bladder cancer. Consequently, current report of the American Association for the Study of Liver Diseases and the European Association for the Study of the Liver suggests the use of pioglitazone/vitamin E for treating biopsy-proven MASH, mentioning that pioglitazone is off-label in patients without T2D and may cause weight gain. Lobeglitazone, another PPARγ agonist, also leads to moderate weight gain and attenuates hepatic steatosis, with an improvement of glucose and lipid homeostasis in patients with T2D; however, more randomized controlled trials must be conducted to further assess the effects of lobeglitazone [109].

### 9.3. PPARα/γ Agonists

Randomized trials tested pioglitazone (PPARγ ligand) and elafibranor (dual PPARα and β/δ ligand) as treatments for MASLD and MASH. However, these drug candidates had significant side effects; thus, further research is needed to determine the optimal dosing regimens of these drugs to achieve the best therapeutic effects with fewer side effects [113,114]. Saroglitazar was designed to act as a PPARα and PPARγ agonist. PPARα agonism enhances FA oxidation in the liver, reduces the synthesis and secretion of TGs, and improves circulating lipoprotein profiles. Activation of PPARγ regulates genes that increase insulin sensitivity and suppress blood glucose and glycosylated hemoglobin A1c levels. Saroglitazar, a PPARα/γ agonist, is an innovative MASLD/MASH therapy that is in an ongoing phase 3 clinical trial. Another example is lanifibranor (IVA-337), a pan agonist, which causes less weight gain and normalizes plasma glucose and insulin levels. The drug is considered promising in improving MASH histology. In addition, IVA337 is more effective in preventing and reversing liver fibrosis than any other agonist [115]. Phase 2 clinical trials have evaluated the efficacy of IVA337 against intrahepatic TGs and MASH (a phase 3 clinical trial is now underway) [116]. These results suggest that dual and pan agonists are promising candidates for MASLD/MASH treatment. 

**Table 1:** Potential of PPAR ligands as therapeutic agents.

PPAR Ligands	Isotype	Status	Source	Reference
Arachidonic acid	α		Nature	[117]
Leukotriene B4	α		Nature	[118,119]
Phosphatidylcholine	α		Nature	[120,121]
Resveratrol	α		Nature	[122,123]
Linoleic acid	γ		Nature	[124]
Prostaglandin D2	γ		Nature	[125]
15-deoxy-delta 12,14-prostaglandin J2	γ		Nature	[126]
Lysophosphatidic acid	γ		Nature	[127]
Clofibrate	α	Clinical	Synthesis	[128]
Fenofibrate	α	Clinical	Synthesis	[129,130]
Bezafibrate	α	Clinical	Synthesis	[131]
Gemfibrozil	α	Clinical	Synthesis	[132]
Pemafibrate	α	Clinical	Synthesis	NCT03350165 [110,133,134,135,136,137,138]
WY14643	α	Basic	Synthesis	[139]
GW9578	α	Basic	Synthesis	[140]
GW7647	α	Basic	Synthesis	[141,142]
Pioglitazone	γ	Clinical	Synthesis	NCT00063622 NCT00062764 NCT00013598 NCT03646292 NCT04976283 NCT04501406
Ciglitazone	γ	Clinical	Synthesis	[143]
Troglitazone	γ	Clinical	Synthesis	[144,145]
Rosiglitazone	γ	Clinical	Synthesis	[146,147,148]
S26948	γ	Clinical	Synthesis	[149]
INT131	γ	Clinical	Synthesis	[150,151]
Saroglitazar	α/γ	Clinical	Synthesis	NCT05011305 NCT03639623 NCT03061721 NCT02265276 NCT03863574 NCT03617263 NCT03112681 NCT04193982
Lobeglitazone	α/γ	Clinical	Synthesis	[152,153]
Lanifibranor (IVA-337)	pan	Clinical	Synthesis	NCT03008070 NCT02503644 NCT05232071 NCT04849728

## 10. NP-Mediated Drug Delivery Systems (NDDSs) as Potential Therapeutic Agents

### 10.1. Nanoparticles System

**Figure 3:**
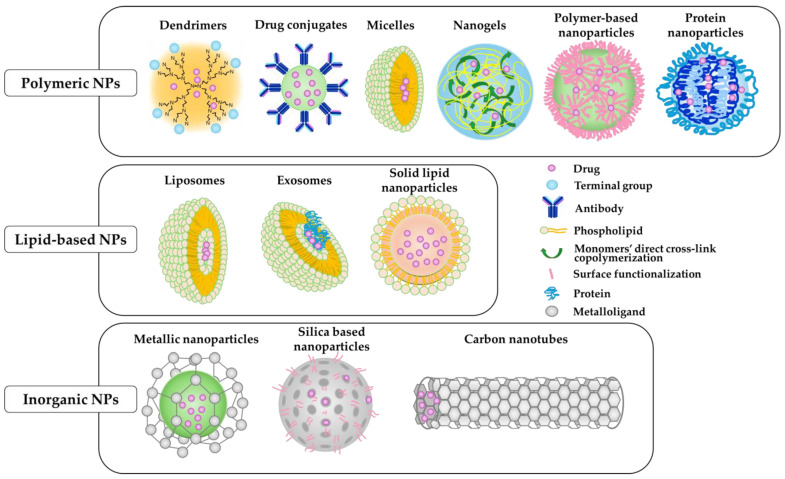
Schematic diagram of the main types of NPs.

Advancements in nanotechnology have led to the development of NPs for clinical treatment and NDDSs. NDDSs are promising tools for drug delivery. A key advantage of NDDS is its ability to target specific sites in the body, thereby reducing the risk of adverse effects by exploiting the unique physiological and pathophysiological properties of diseases (Table 2) [154]. NPs are ultrafine particles that typically range from 1 to 100 nm in size and possess beneficial physicochemical attributes, including small size, shape, surface charge, hydrophobicity, and chemical composition. NPs exist in various forms, including lipid-based, polymeric, and inorganic NPs [155,156,157] (Figure 3).

Lipid-based NPs, including liposomes, micelles, and wrapsomes, are spherical vesicles comprising one or more phospholipid bilayers that spontaneously assemble in aqueous environments. Their lipid composition, akin to cellular membranes, facilitates enhanced cellular uptake. In contrast to conventional drug delivery methods, lipid-based systems offer reduced toxicity and permit higher drug dosages. Moreover, their amphipathic nature enables interactions with and transport of hydrophilic and hydrophobic compounds, either encapsulated within the NP core or embedded within the lipid bilayer. Polymeric NPs encompass those synthesized from synthetic polymers, such as poly (D-1-lactic acid) and poly (D-L-lactic co-glycolic acid) or natural polymers, such as chitosan and collagen. The vast array of polymer combinations yields unlimited variations in composition and properties, facilitating drug encapsulation within the polymer matrix or display on its surface. Inorganic NPs, such as iron oxide NPs containing iron cores in different oxidation states (magnetite and maghemite), are encased in hydrophilic layers of dextran or other biocompatible components. These coatings enhance stability and functions and reduce cytotoxicity.

### 10.2. Mechanisms of NPs in Targeting Liver Disease

Based on the advantages of NPs, drug delivery can proceed through either passive targeting by sizes and shapes or active targeting by modifying NP surfaces that are similar to those on ligands that are suitable for different cells. Along with these features, NPs also have a promising perspective for treating liver diseases, including MASLD and MASH.

#### 10.2.1. Passive Targeting

**Figure 4:**
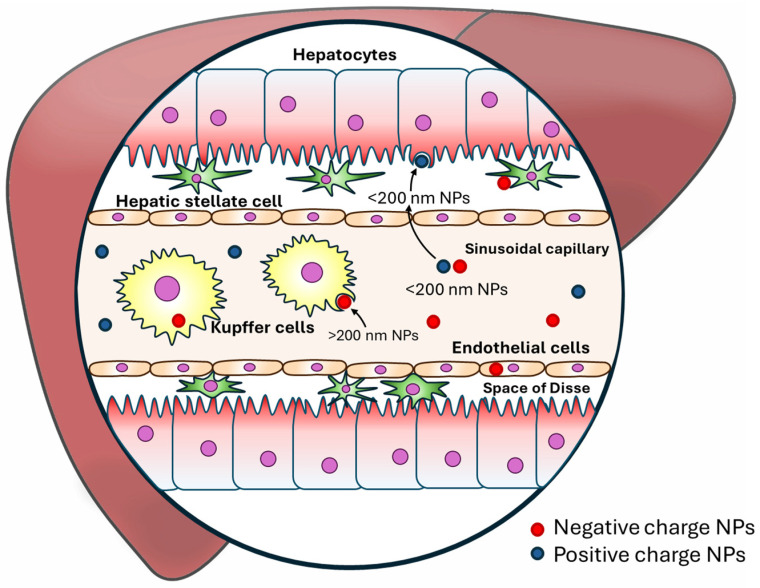
Passive targeting nanoparticles mechanism.

Passive delivery depends on the sizes and special surface modifications of NPs, which can be delivered to specific cells via oral, intravenous, or intramuscular administration and intake by phagocytosis (NPs < 500 nm), pinocytosis, or receptor-mediated endocytosis specifically, negatively charged NPs are more specialized for KCs or endothelial cells, and positively charged or NPs with diameters < 200 nm are uptaken by hepatocytes without macrophage retention (Figure 4) [158,159,160]. Further reports also showed that different NP shapes could affect the effectiveness of specific cell-targeting treatments. For instance, rod-shaped and worm-like NPs are less captured by KCs than spherical-shaped NPs [161,162]. In addition, hydrophobic NPs are filtered out of the blood by the reticuloendothelial system faster than hydrophilic NPs; hence, NPs are usually polyethylene glycol (PEG)ylated to reduce protein binding and allow them to circulate longer in the blood.

The size and shape advantages of NPs allow the intentional targeting of specific cells at various stages of liver disease progression. 

#### 10.2.2. Active Targeting

In contrast to passive targeting, active targeting uses specific components for conjunction, such as antibodies, proteins, peptides, aptamers, carbohydrates, and vitamins that will target a receptor on the cell’s membrane [163,164].

The liver is a complex vital organ composed of two primary cell types: parenchymal and non-parenchymal cells. Most metabolic functions of the liver are performed by hepatocytes and cholangiocytes, which constitute 60–70% of the parenchymal cells. The remaining 30–40% of liver cells are non-parenchymal cells, including LSECs, HSCs, and KCs, that play essential roles in the liver’s physiological processes. The development of cell-specific transport systems is necessary to enhance drug efficacy and reduce drug toxicity (Table 3). 

Hepatocytes have a major role in MASLD and MASH progression; thus, hepatocyte-targeting NPs are meaningful. Targeting hepatocytes for drug delivery is challenging due to the lack of specific surface receptors for the binding of antibodies or peptides. However, this problem can be solved by mimicking natural nanocomplexes, such as lipoproteins, to create hepatocyte-directed nanomedicines. Lipopeptide NPs show remarkable efficiency in targeting and delivering siRNAs to hepatocytes in vivo [165]. This delivery system is more efficient than non-parenchymal cells and exhibits the potential to revolutionize the treatment of liver diseases. Asialoglycoprotein receptors and glycyrrhizin, which are hepatocyte-specific ligands, are used for targeted drug delivery in vivo [166,167,168]. pH-labile PEGylated lipid NPs with N-acetylgalactosamine modifications interact with asialoglycoprotein receptors for active liver targeting [169]. This approach also facilitated the targeted delivery of Lactosylated-poly 2-dimethylaminoethyl methacrylate NPs in mouse models of MASLD, reducing disease pathology [170]. Another drug delivery method takes advantage of GL, a hepatocyte-specific ligand, to target hepatocytes in vivo. As a result, it showed that NPs containing a GL modification increased efficient uptake by hepatocytes [168]. Several studies have shown that this strategy has a high hepatocyte transfection rate (up to 90%) [171].

To target HSCs, the primary drivers of fibrosis in MASLD/MASH, several studies have modified the surface receptors of HSCs or specific ligands of highly expressed receptors, such as mannose-6-phosphate/insulin-like growth factor II (M6P/IGF-II) [172], PDGF receptor [173], and retinol-binding protein receptor, on the surface of NPs to selectively deliver antifibrotic drugs to HSCs [174]. HSCs are the primary vitamin A-storing cells in the body; therefore, coating NPs with vitamin A directs them to HSCs via interactions between vitamin A and retinol-binding protein receptors. Administration of siRNA-loaded lipid NPs containing vitamin A significantly reduces collagen production in mice with liver fibrosis [175]. Vitamin A-coupled liposomes (VA-lip-siRNAgp46) have been developed to target HSCs involved in fibrosis. Rats administered VA-lip-siRNAgp46 exhibit markedly higher survival rates than those administered control liposomes without vitamin A modification [176]. NPs exhibit higher entry into HSCs compared to the controls, leading to increased survival rates in rats with fibrosis. Furthermore, hepatic silybin levels in animals treated with vitamin-loaded lipid NPs are 2–3 times higher than those in animals treated with control lipid NPs and free silybin levels in vivo [177]. M6P-modified liposomes have been tested for their targeting efficiency toward HSCs in vitro and in vivo. The uptake of M6P-modified liposomes by activated HSCs is four times higher than that of unmodified liposomes [178]. Recently, PDGF-β, overexpressed in activated HSCs, was targeted using pPB peptide-coated nucleic acid-lipid NPs to deliver siRNAs for hepatic fibrosis treatment [179]. Another example is dual-functional NPs, designed to target HSCs, aimed to reduce fibrosis and angiogenesis in a mouse model, incorporating a C-X-C motif chemokine receptor 4 antagonist to release siRNA against vascular endothelial growth factor [180].

The development of an NDDS targeting KCs presents a viable approach to combat inflammation and liver injury associated with MASLD/MASH. Receptors expressed on KCs, such as mannose and scavenger receptors, are used to deliver anti-inflammatory drugs, ROS scavengers, phenotype-transforming agents, and siRNA drugs to the target cells [181]. In vivo experiments with mannose-modified trimethyl chitosan-cysteine conjugated NPs revealed effective inhibition of TNF-α production in liver macrophages. Moreover, they reduce the degree of liver injury and fibrosis in mouse models and protect them from liver damage and inflammation-induced lethality. Another study reported NDDSs as viable options for liver fibrosis treatment [182]. In addition, phosphatidylserine signals phagocytosis, enhancing uptake by hepatic macrophages [183]. Thus, phosphatidylserine-modified nanostructured lipid carriers were developed to prolong the retention of curcumin, increasing its bioavailability and distribution in vivo, which resulted in reduced liver fibrosis and inflammation [184]. NP drugs targeting LSECs, the key inducers of liver injury, inhibit tumor growth by recruiting natural killer T cells using simvastatin, a nanodrug, converting HSCs from activation to quiescence state for the expression of C-X-C motif chemokine ligand 16 on LSECs [185]. Functionalizing polymeric micelles loaded with simvastatin target LSECs to preserve liver function during chronic liver disease [186]. Titanium dioxide NPs restore sinusoidal permeability via the induction of leakiness in primary human LSECs, thus promoting liver recovery and enhancing drug uptake [187], highlighting the potential of inorganic NPs as novel tools to promote drug delivery to the diseased liver. 

Nanotechnology can revolutionize the treatment of MASLD/MASH. Nanomedicine offers more effective and targeted therapies, ultimately improving the outcomes of patients with liver diseases.

**Table 3:** Nanotechnology applications for the therapy of liver disease.

Types of Nanoparticles	Nanoparticle Formulation	Drug	Target Cell	Impact on Liver Diseases	Reference
Polymeric NPs	PLGA-PEG-Mal	Nilotinib	HSC	Efficiently degrading pericellular collagen I showed optimal antifibrotic activity, liver fibrosis therapy	[188]
	Diblock copolymers poly[oligo(ethylene glycol) methyl ether methacrylate]-block-VDM	S-nitrosoglutathione	HSC	Alleviating both liver fibrosis and portal hypertension	[189]
	PEG-PLGA	Sorafenib	HSC	Efficiently ameliorated liver fibrosis by decreased alpha-smooth muscle actin (α-SMA) content and collagen production in the livers of CCl4-treated mice, shrank the abnormal blood vessels, and decreased microvascular density	[190]
	Retinol–chitosan NPs	JQ1- and atorvastatin	HSC	Preventing HSC activation showed reduction in α-SMA expression	[191]
	Monomethoxy-PEG-PLGA	Rapamycin	Hepatocyte	Improve hepatic steatosis and liver injury in MASLD	[192]
	Polyurethane NPs	Fenofibrate	Hepatocyte	Inhibitory effects on MASLD	[193]
	Chondroitin sulfate nanomicelles codelivery system	Retinoic acid and doxorubicin	HSC, Hepatocyte	Destroyed the Golgi structure and ultimately downregulated COL1 production	[194]
	Cyclic RGD peptides-PEG-PLGA	miR-29b and germacrone	HSC	Co-delivery of miR-29b and germacrone based on Cyclic RGD peptides-modified NPs for liver fibrosis therapy	[195]
	Poly(beta-amino-ester) (PBAE) 536	DNA labeled with a fluorescent marker	Hep3b	Theranostic gene delivery to HCC	[196]
Lipid-based NPs	pRelaxin-Lipid protamine DNA	Aminoethyl anisamide	HSC	Deactivated HSC, macrophage phenotype switch, the relaxin-primed alleviation of liver fibrosis	[197]
	cRGDyK-guided liposomes	The hedgehog inhibitor Vismodegib	HSC	Preferentially internalized by activated HSCs in vitro and in vivo, deliver therapeutic agents to activated HSC to treat liver fibrosis	[198]
	Asialoglycoprote-liposomes loaded	Norcantharidin	HepG2	Effectively reduce renal and hepatocellular toxicity	[199]
	Vitamin A-coupled liposomes	siRNA against a collagen-specific chaperone	HSC	Effective in suppressing collagen secretion and treating fibrosis induced by CCl4 or bile duct ligation	[176]
Inorganic NPs	Gold nanorods-PEG-PDGFRβ		HSC	Decreased fibrosis, hepatic inflammation, and hepatocyte injury	[200]
	Calcium phosphate NPs	Tumor necrosis factor-stimulated gene 6	Macrophages	Tumor necrosis factor-stimulated gene 6 exerts an antifibrotic effect by facilitating the transition of macrophages to the M2 phenotype and by enhancing the expression of matrix metalloproteinase-12	[201]

## 11. Discussion and Conclusions

The MASLD/MASH includes various processes such as lipid metabolic disorders, oxidative stress, intracellular inflammatory responses, and ER stress. Lipid metabolic disorders play a key role in triggering MASLD/MASH progression, causing inflammation, cell death, and fibrogenesis. PPARα and PPARγ are important regulators of lipid homeostasis and insulin sensitivity in the liver, significantly influencing MASLD/MASH progression. Recent single-nuclei RNA sequencing data have revealed their expression in all liver cells, with PPARα highest expression in hepatocytes and cholangiocytes and PPARγ in liver macrophages and LSECs. PPARα and PPARγ functions have been well studied in hepatocytes and macrophages but not cholangiocytes and LSECs, respectively. Their understanding may be important to achieve high therapeutic efficacy. 

Therapeutic interventions using PPARα and PPARγ agonists have shown efficacy in treating MASLD and MASH. Preclinical and clinical studies indicate that PPARα agonists, such as Wy-14643, prevent TG accumulation, while PPARγ agonists, like pioglitazone, exhibit anti-inflammatory and antifibrotic effects. In addition, combining PPARγ activation with TGF-β pathway suppression could offer a synergistic approach to inhibit liver fibrosis, a key feature of MASLD [202]. Similarly, pairing classical antioxidants like vitamin E with PPARγ agonists might delay MASLD progression by enhancing the scavenging of free radicals and reducing oxidative stress [203]. MASLD/MASH often impairs the effectiveness of immunotherapy due to aberrant T cell activation and the subsequent activation of HSCs [204]. Considering the role of PPARγ in regulating hepatic inflammatory responses, combining PPARγ modulators with immune checkpoint inhibitors could be an effective strategy for MASLD/MASH-related disorders, including HCC. Future perspectives could focus on developing strategies that combine PPAR with other factors to better manage MASLD/MASH. However, because drug development of these agonists is often halted due to side effects, the development of cell-specific DDS is necessary. Nanotechnology breakthroughs have provided new possibilities for efficient DDS. NPs have many advantages that increase the efficiency of treatment with minimized side effects and the capability of selective targeted distribution. Targeting the liver cells such as HSCs, LSECs, and macrophages showed promising results. NP-based drugs facilitate liver disease therapies and hold great promise for future applications, especially for MASLD/MASH treatment. However, further optimization for NPs delivery systems is required according to their complexity and interactions with liver cells.

NPs show perspective results at the early stage of development with better outcomes that can change the state of affairs in liver disease therapies and improve the life quality of patients in the future. The specialized PPARα- and PPARγ-targeted NP drugs will be excellent solutions for MASLD/MASH treatment.

## Figures and Tables

**Table 2:** Advantages and disadvantages of various NPs used as drug delivery systems.

Types of Nanoparticles	Advantages	Disadvantages
Polymeric NPs	Generally, excellent stability Controlled synthesis and a great deal of flexibility in conjugation of multiple bioactive agents Tailored drug release pattern Flexibility in administration routes	Agglomeration Nanotoxicity with non-biodegradable polymers
Lipid-based NPs	Enhances the solubility of hydrophobic therapeutic agents, ensuring high bioavailability and biodistribution Effective drug protection and controlled release, encapsulating both polar and non-polar bioactive agents Engineered for targeting specific sites, it offers enhanced stability and boasts a longer shelf life The ingredients used are biocompatible and biodegradable, leading to high cell uptake and effective drug protection in acidic pH With the ability to penetrate deeply, good stability, easy drug entrapment, and sustained release of the entrapped drug, ensuring long-lasting therapeutic effects	Not crossing the stratum corneum barrier, rigid structure Drug leakage, particle aggregation, high production costs, and the scarcity of Food and Drug Administration-approved polymers Highly prone to oxidative degradation, high cost, and impurity of natural phospholipids Gelling tendency Optimization required of the ratio of solid/liquid lipids Low stability Some may be allergic
Inorganic NPs	Biocompatible Ease of synthesis and conjugation of multiple bioactive agents Control of particle size Ease of synthesis and conjugation of multiple bioactive agents Large surface area Ability to encapsulate and deliver various types of bioactive agents Protects entrapped drug and provides sustained release	Not biodegradable High cost of large-scale production Poorly soluble in water Toxicity concerns

## Data Availability

All data used to support this work are included in the article.

## Abbreviations

ACAD, Acyl-CoA dehydrogenase; ACAT, Acyl CoA cholesterol acyltransferases; ACC, Acetyl-CoA carboxylase; ADIPOQ, Adiponectin; APOA1, Apolipoprotein A1; APOA2 Apolipoprotein A2; ATGL, Adipose triglyceride lipase; CD, Cluster of differentiation; CEs, Cholesteryl esters; CGI, Comparative gene identification; ChREBP, Carbohydrate response element-binding protein; COL1A1, Collagen type I alpha 1; DGAT, Diacylglycerol-O-acyltransferases; ER, Endoplasmic reticulum; FAS, Fatty acid synthase; FA, Fatty acid; Fsp27, Fat-specific protein 27; GL, Glycyrrhizin; HCC, Hepatocellular carcinoma; HSCs, Hepatic stellate cells; IL, Interleukin; IR, Insulin resistance; KCs, Kupffer cells; LPL, Lipoprotein lipase; LPS, Lipopolysaccharide; LSECs, Liver sinusoidal endothelial cells; M6P/IGF-II, Mannose-6-phosphate/insulin-like growth factor II; MASLD/MASH, Metabolic dysfunction-associated steatotic liver disease/steatohepatitis; MCD, Methionine and choline-deficient; miR, MicroRNA; NDDS, NP-mediated drug delivery systems; NF-κB, Nuclear factor kappa B; NLRP3, NLR family of pyrin domain containing 3; NPs, Nanoparticles; PDGF, Platelet-derived growth factor; PLGA, Poly (lactic-co-glycolic acid); POEGMA, Poly[oligo(ethylene glycol) methyl ether methacrylate]; PPARs, Peroxisome proliferator-activated receptors; ROS, Reactive oxygen species; SCD1, Stearoyl-CoA desaturase 1; SMAD, Mothers against decapentaplegic homolog; SREBP-1C, Sterol regulatory element-binding protein 1C; TFEB, Transcription factor EB; TGF-β1, Transforming growth factor beta 1; TGs, Triglycerides; TIMP, Tissue inhibitor of metalloproteinase, TLRs, Toll-like receptors; TNF-α, Tumor necrosis factor alpha; TZD, Thiazolidinedione; T2D, Type 2 diabetes; VLDL, Very-low-density lipoprotein; α-SMA, Alpha-smooth muscle actin.
